# Fentanyl-contaminated drugs and non-fatal overdose among people who inject drugs in Baltimore, MD

**DOI:** 10.1186/s12954-018-0240-z

**Published:** 2018-07-05

**Authors:** Ju Nyeong Park, Brian W. Weir, Sean T. Allen, Patrick Chaulk, Susan G. Sherman

**Affiliations:** 1 Department of Health, Behavior and Society, Johns Hopkins Bloomberg School of Public Health, Ju Nyeong Park, 624 N Broadway, HH163, Baltimore, MD 21205 USA; 2 Department of Epidemiology, Johns Hopkins Bloomberg School of Public Health, 615 N. Wolfe St, Baltimore, MD 21205 USA; 3 Baltimore City Health Department, 1001 E. Fayette St, Baltimore, MD 21201 USA; 4 Department of Health, Policy and Management, Johns Hopkins Bloomberg School of Public Health, 624 N. Broadway, Baltimore, MD 21205 USA; 5 Division of Infectious Diseases, School of Medicine, Johns Hopkins University, Baltimore, MD 21205 USA

**Keywords:** Opioids, Heroin, Overdose, Substance use, Epidemiology

## Abstract

**Background:**

The opioid crisis remains a major public health issue in the US and beyond. Despite rapid rises in fentanyl-related mortality nationally, little is known about the role of fentanyl in the occurrence of non-fatal overdose among people who use drugs. We examined the prevalence of non-fatal overdose and perceived fentanyl exposure among syringe services program (SSP) clients and modeled the correlates of non-fatal overdose.

**Methods:**

Data were drawn from a cross-sectional survey of 203 SSP clients in Baltimore, MD recruited in 2016. Logistic regression models were used to identify the correlates of experiencing non-fatal overdose in the past 12 months.

**Results:**

The majority (65%) was male, 52% were black, 41% were white, and 37% were homeless. Almost all (97%) used heroin, 64% injected heroin with cocaine (i.e., speedball), and many used other types of drugs. Half (53%) perceived fentanyl presence in their drugs either half, most or all of the time. Lifetime and past 12 month prevalence of non-fatal overdose were 58 and 31%, respectively. Independent correlates of non-fatal overdose in the past 12 months were perceiving fentanyl in drugs more than half the time (aOR = 2.79; 95% CI = 1.00–4.68), speedball injection (aOR = 2.80, 95% CI = 1.26–6.23), non-prescription buprenorphine use (aOR = 6.37; 95% CI = 2.86–14.17), and homelessness (aOR = 3.07; 95% CI = 1.28–7.39).

**Conclusions:**

These data demonstrate that SSP clients are at high-risk of overdose, some of which is likely attributable to fentanyl exposure. Addressing the rising fentanyl epidemic will require comprehensive and innovative strategies that attend to drug use patterns and structural factors such as homelessness.

## Background

Opioid overdose was the leading cause of injury-related death in the US in 2015, surpassing homicides. This crisis has claimed almost half a million lives since 2000 in the US alone [1]. Over the last two decades, the opioid overdose epidemic has been largely driven by non-medical prescription opioid use and heroin use [1]; however, in recent years, dramatic increases in deaths have been attributable to the rise of synthetic opioids in the illegal drug market. Fentanyl, a synthetic opioid 50–100 times more potent than morphine, is difficult to dose correctly and is associated with high mortality rates, particularly in the Northeast [1–3]. Drug seizure data from the Drug Enforcement Administration (DEA) has shown that although pure pharmaceutical fentanyl can be diverted from medical use, fentanyl is often manufactured illicitly, and is found in street drugs such as white powder heroin and cocaine, thus increasing the lethality of these drugs [4]. A recent study of harm reduction service clients in British Columbia, Canada, found that most people who use drugs who test positive for fentanyl are unaware that their drugs contain fentanyl [5].

People who inject drugs (PWID) are at highest risk of experiencing and witnessing an overdose. Non-fatal overdose is a strong risk factor for future overdose (fatal and non-fatal) and is associated with a range of health risks including cognitive impairment and muscular dysfunction as well as high healthcare costs [6, 7]. Other correlates of non-fatal overdose include “polysubstance use” (i.e., the use of multiple types of substances that can act synergistically to increase overdose risk, such as the concomitant use of opioids and alcohol, or opioids and benzodiazepines), homelessness, injecting in public spaces (e.g., streets or abandoned houses), and police encounters [8–14]. However, the role of fentanyl in non-fatal overdose is an understudied issue in the published literature.

Baltimore City, like many parts of the US, is in the midst of an opioid overdose epidemic. From 2015 to 2016 in Baltimore City, overdose mortality related to heroin increased by 75%, from 260 to 454, and fentanyl-related overdose mortality increased by a staggering 249% from 120 to 419 [15]. In one recent survey of illicit opioid users in Rhode Island, a US state also located in the Northeast, about half reported exposure to fentanyl in the past year, and more frequent heroin use was associated with self-reported fentanyl exposure [16]. In recognition of this burgeoning epidemic, we measured the prevalence of self-reported fentanyl-contamination among a sample of Baltimore Syringe Services Program (SSP) clients who predominantly injected heroin, and examined the correlates of non-fatal overdose among this population. The study was conducted at the Baltimore City SSP, one of the first publically funded SSPs in the US, which has conducted overdose education and naloxone distribution to their clients since 2004 [17].

## Methods

Data collection for this cross-sectional study occurred between April and November 2016. Recruitment occurred through targeted sampling with all SSP sites (*n* = 16) included in the sampling frame and recruitment targets at each site weighted by total client size per site. Study staff approached clients after they exited the SSP van and briefly explained the study, conducted a brief screening and invited eligible clients to participate in a 30-min interviewer-administered computer-assisted personal interview survey. Eligibility criteria included being a registered SSP client and being at least 18 years of age. Informed consent was given orally, and study participation was anonymous. The survey instrument ascertained socio-demographics, housing status, police interactions, drug use behaviors, perceptions of fentanyl contamination, drug treatment, and experiences surrounding overdose, overdose response training and naloxone use. Participants were compensated with a pre-paid $25 VISA card. The study was approved by the Johns Hopkins Bloomberg School of Public Health Institutional Review Board.

### Measures

The primary outcome of interest was non-fatal overdose in the past 12 months (yes/no), which was constructed from the following question: “Have you ever experienced an overdose?” and, if yes, “When was the last overdose?” Response options were within last week, month, 6 months, a year, or more than a year ago. Race/ethnicity was collapsed into a binary variable for multivariate analysis (non-Hispanic White vs. Hispanic, Black, African American, multiracial, or other). Housing status was captured using “In the last 3 months, where did you usually sleep at night?” with responses grouped into three categories: own/rent a house or apartment vs. staying with family/friends vs. homeless (on the streets, in a car, in an abandoned house, or at a shelter).

Self-reported fentanyl contamination was captured with the question, “When you inject drugs, how often do you think it is laced with fentanyl?” Responses were categorized for regression analysis (never/rarely vs. about half the time/most of the time/always vs. do not know). Drug injection frequency was measured using the question “In the past 6 months, how often did you inject any drug?” (daily or more/more than once a week/once a week/more than once a month/once a month/less than once a month/never). These responses were collapsed into a binary variable: daily or more (yes/no). The survey also measured the average number of times the respondent injected on a given day (once/2–3 times/more than 3 times). This variable was collapsed into a binary variable: injected > 3 times a day (yes/no). Binary (yes/no) variables for injection and non-injection drug use in the past 6 months were constructed using “When was the last time you [insert route of administration] [insert drug type].” The survey also asked about places in the community where drugs are injected using two items “In the last 30 days did you inject in the following places?” (own home/somebody else’s home/abandoned building/street or park/vehicle/shooting gallery/public bathroom/other) and “Of those places you injected in the last 30 days, where did you inject the most?” Response options to the latter item were grouped into private (own home or somebody else’s home) vs. public (abandoned building, street, park, vehicle, shooting gallery, public bathroom, and other) for regression analysis.

### Statistical analysis

The analytic sample was limited to participants who had injected drugs in the past year, resulting in the exclusion of one participant. Prevalences were calculated for socio-demographics, non-fatal overdose, witnessing overdose, overdose training uptake, naloxone use, thoughts on whether drugs contain fentanyl, and injection drug use behaviors. In order to examine the factors associated with recent non-fatal overdose, bivariate logistic regression models of the covariates were run, and the sub-set of correlates significant at the *p* < 0.20 level were reported and considered for inclusion in multivariable logistic regression modeling. Backwards stepwise selection with log likelihood testing was used with variables at *p* ≥ 0.15 removed. The Akaike information criterion and Bayesian information criterion were considered in selecting the final model. A post hoc Pearson’s chi-square test was used to test the association between homelessness and public injection due to collinearity. Public injection was strongly associated with homelessness, and both variables were associated with non-fatal overdose; however, homelessness held a stronger association with the outcome and therefore was retained in the final multivariable model after checking the AIC and BIC of both models. All analyses were conducted in Stata/SE 14.2 (College Station, TX).

## Results

About half (54%) of participants (*N* = 203) were ages 45 and over, the majority (65%) were male, 52% were non-Hispanic black, 41% were non-Hispanic white, and 62% had graduated from high school/GED (Table 1). The prevalence of homelessness (excluding those staying with family/friends/other people) was 37%, and the prevalence of being stopped by police within 2 or 3 blocks around the SSP past 6 months was 23%. Many (36%) PWID had been arrested or incarcerated in the past 12 months.

**Table 1:** Socio-demographic and structural characteristics of people who inject drugs in Baltimore, Maryland (*N* = 203)

	Number	Percent
Age		
18–34	48	23.7
35–44	45	22.2
45–54	70	34.5
≥ 55	40	19.7
Sex		
Male	132	65.0
Female	71	35.0
Race/ethnicity		
Non-Hispanic Black	105	51.7
Non-Hispanic White	83	40.9
Hispanic/multi-race/other	15	7.4
Educational attainment		
Less than high school	77	37.9
12th grade or GED	90	44.3
College, some college, associate’s/technical degree	36	17.7
Housing status, past 3 months		
Own or rent a house/apartment	79	38.9
Staying with family or friends, or other	50	24.6
Homeless^#^	74	36.5
Stopped by police within 2 or 3 blocks around NEP, past 6 months	46	22.8
Arrested, past 12 months	73	36.0

^#^Streets, car, abandoned houses, shelter, no set place, or do not know

*GED* General Educational Development, *NEP* needle exchange program

About half (53%) thought their drugs contained fentanyl between half to all the time, and 17% were unsure whether their drugs contained fentanyl. The majority (93%) injected heroin by itself, or with cocaine (i.e., speedballing) (65%), and many injected cocaine alone (58%) (Table 2). Prevalence of injecting prescription opioids in the past 6 months was 12%. About half (55%) smoked crack cocaine, 37% snorted/smoked heroin, 29% swallowed or snorted prescription opioids, 27% misused buprenorphine, and 31% misused benzodiazepines. A third (33%) had been in an outpatient drug treatment program in the past 12 months. Inpatient hospital-based drug treatment and inpatient rehabilitation were less common (8 and 7% respectively). Sixty percent injected drugs mainly in a private residence compared to 40% who injected mainly in a public place.

**Table 2:** Perceived fentanyl contamination, drug use, and drug treatment among people who inject drugs in Baltimore, MD (*N* = 203)

	Number	Percent
Perceived fentanyl contamination		
“When you inject drugs, how often do you think it is laced with fentanyl?”		
Never or rarely	60	29.9
Half to all the time	107	53.2
Do not know	34	16.9
Drug use, past 6 months		
Injected:		
Heroin alone	188	93.1
Speedball (heroin and cocaine)	132	65.4
Cocaine alone	117	57.9
Prescription opioids	25	12.4
Smoked crack cocaine	112	55.2
Snorted/smoked heroin	74	36.6
Swallowed or snorted prescription opioids*	59	29.2
Swallowed buprenorphine/Suboxone*	54	26.6
Swallowed benzodiazepines*	62	30.7
Drug treatment, past 12 months		
Outpatient treatment	66	32.5
Inpatient hospital	17	8.4
Inpatient rehabilitation	16	7.9

*Misuse, i.e., taken not as prescribed or without a prescription

Overdose was a common occurrence among our sample of PWID. Over half (58%) had ever experienced an overdose, almost a third (31%) had overdosed in the past 12 months, and the majority (90%) had witnessed an overdose (Table 3). The majority (79%) had ever received overdose response training, and 44% had ever used naloxone. When used, naloxone administration successfully reversed the overdose 99% of the time. Overall, 42% of PWID did not currently have naloxone either with them or at home. Among PWID who had received naloxone at an overdose response training, 29% (45/153) did not currently have naloxone either with them or at home.

**Table 3:** Non-fatal overdose, witnessing an overdose, overdose training, and naloxone use among people who inject drugs in Baltimore, MD (*N* = 203)

	Number	Percent
Experienced non-fatal overdose		
Ever	117	57.6
Past 12 months	63	31.0
Naloxone administered at last overdose	34	54.0
Went to emergency room at last overdose	23	36.5
Witnessed overdose		
Ever witnessed overdose	182	89.7
Ever witnessed non-fatal overdose	166	81.8
Ever witnessed fatal overdose	77	37.9
Received overdose response training		
Ever	158	77.8
Trained by Baltimore City Health Department	138	87.3
Past 12 months	146	71.9
Use of naloxone		
Ever used	90	44.3
Without overdose training	11	12.2
Successful during most recent time used	89	98.9
Currently have naloxone on person or at home	117	58.2

Unadjusted bivariate logistic regression analysis (Table 4) revealed increased odds of recent non-fatal overdose among PWID who thought that their drugs contained fentanyl at least half of the time (OR = 2.48, 95% CI = 1.18–5.22), were non-Hispanic White (OR = 2.20, 95% CI = 1.19–3.99), homeless (OR = 2.94, 95% CI = 1.45–5.95), injected speedball (OR = 2.03, 95% CI = 1.03–3.97), misused prescription opioids in the past 6 months (OR = 2.34, 95% CI = 1.00–5.49), misused non-prescription buprenorphine (OR = 4.39, 95% CI = 2.27–8.51), used public spaces as main place of injection (OR = 2.07, 95% CI = 1.12–3.82), or accessed inpatient hospital-based drug treatment (OR = 3.58, 95% CI = 1.30–9.91). Post hoc unadjusted analysis demonstrated that homelessness in the past 3 months was strongly associated with using public spaces as the main place of injection in the past 30 days (χ^2^ (2, 196), *p* < 0.001).

**Table 4:** Socio-demographic, behavioral, and structural correlates of non-fatal overdose in the past 12 months among people who inject drugs in Baltimore, MD (*N* = 202)

	OR	95% CI	*p*	aOR	95% CI	*p*
“When you inject drugs, how often do you think it is laced with fentanyl?”						
Never or rarely	REF	–	–	REF	–	–
Half, most, or all the time	2.48	1.18–5.22	0.016	2.79	1.00–4.68	0.021
Do not know	1.38	0.52–3.72	0.518	0.89	0.28–2.82	0.840
Female gender	1.29	0.69–2.40	0.421	2.16	1.00–4.68	0.050
Race/ethnicity						
Black, Hispanic, or other	REF	–	–	–	–	–
Non-Hispanic White	2.20	1.19–3.99	0.012	–	–	–
Housing status, past 3 months						
Own/rent	REF	–	–	REF	–	–
Staying with family or friends or other	1.28	0.56–2.94	0.558	1.41	0.54–3.68	0.483
Homeless^#^	2.94	1.45-5.95	0.003	3.07	1.28–7.39	0.012
Arrested, past 12 months	1.69	0.92–3.11	0.092	–	–	–
Injected > 3 times per day	1.73	0.91–3.29	0.095	–	–	–
Injected heroin by itself, past 6 months	0.41	0.14–1.23	0.114	0.13	0.03–0.49	0.002
Injected speedball, past 6 months	2.03	1.03–3.97	0.040	2.80	1.26–6.23	0.012
Prescription opioid misuse,*past 6 months	2.34	1.00–5.49	0.049	–	–	–
Benzodiazepine misuse,*past 6 months	1.81	0.97–3.40	0.064	–	–	–
Buprenorphine/Suboxone misuse,*past 6 months	4.39	2.27–8.51	< 0.001	6.37	2.86–14.17	< 0.001
Main place where drugs injected, past 30 days						
Private residence (own home, someone else’s home)	REF	–	–	–	–	–
Public (e.g., abandoned building, street, park, car, shooting gallery)	2.07	1.12–3.82	0.021	–	–	–
Drug treatment in the past 12 months						
Inpatient hospital	3.58	1.30–9.91	0.014	–	–	–
Inpatient rehab	2.40	0.86–6.72	0.095	–	–	–
Outpatient treatment	0.61	0.32–1.19	0.148	–	–	–

Adjusted logistic regression model is adjusted for age

^#^Streets, car, abandoned houses, shelter, no set place or do not know

*Taken not as prescribed or without a prescription

*OR* odds ratio, *aOR* adjusted odds ratio, *CI* confidence interval, *REF* reference category

In age-adjusted multivariable logistic regression analysis (Table 4), factors independently associated with recent non-fatal overdose were thinking that their drugs contained fentanyl at least half of the time (aOR = 2.79; 95% CI = 1.00–4.68), homelessness (aOR = 3.07, 95% CI = 1.28–7.39), injecting heroin by itself (aOR = 0.13, 95% CI = 0.03–0.49), speedball injection (aOR = 2.63, 95% CI = 1.23–5.64), and buprenorphine misuse (aOR = 4.97, 95% CI = 2.37–10.43). Gender was marginally (*p* = 0.050) associated. No significant multivariable associations were observed for staying in a family or friend’s house/apartment compared to owning/renting (aOR = 1.41; 95% CI = 0.54–3.68).

## Discussion

Overdose has been a chronic public health issue in Baltimore and many parts of the US for several decades, and continues to disproportionately affect PWID. Understanding the correlates of non-fatal overdose—a key risk factor for fatal overdose—is critical in preventing overdose and designing interventions to address the harms associated with heroin and other illicit opioid use. In our examination of the correlates of non-fatal overdose among Baltimore City SSP clients, consisting primarily of non-Hispanic Black and White heroin users, we observed health promotion behaviors including the uptake of naloxone trainings and drug treatment participation juxtaposed against overdose risk behaviors (e.g., speedball injection), as well as structural risk factors such as homelessness, which is consistent with the existing literature. A novel finding was that perceived fentanyl contamination in drugs was associated with recent non-fatal overdose. This finding signals the importance of developing innovative solutions to combating the inevitable supply-side changes that have and will occur in the illicit and unregulated drug market. While this study focused on the perception of the presence of fentanyl contamination, a potent synthetic opioid currently causing the majority of overdose deaths in Baltimore, we note that other types of synthetic opioids and fentanyl analogues will continue to emerge in the drug market, which highlights the increased need for surveillance of the illicit drug supply.

This study occurred during a period of time that saw dramatic concurrent increases in the local and national supply of fentanyl and fentanyl-related overdose deaths [4]. This likely explains the high prevalence of self-reported (though unverified) fentanyl contamination of drugs, and the association with non-fatal overdose in our study, which mirror local increases in fatal overdose attributable to fentanyl [15]. Fentanyl’s high potency, rapid uptake, and strong receptor affinity compared to heroin and morphine may render harm reduction strategies such as using less drugs or responding with a regular dose of naloxone less effective in responding to an overdose [16, 18]. It is clear that the fentanyl era of the opioid epidemic necessitates innovative strategies. Early detection and reporting of new potent synthetic opioids is needed in the context of an unregulated drug market [19]. In one Canadian study, almost three quarters of people who use drugs who had their drugs checked at harm reduction services were unaware of fentanyl presence in their drug supply [5]. Real-time drug checking programs that allow people who use drugs to quickly test their street drugs for `specific potent chemicals, such as fentanyl and its analogues, may be useful in objectively confirming the presence of these chemicals, particularly in areas where fentanyl surveillance data is not available in a timely manner. These services have been implemented in Europe as early as 2001, mostly inside dance raves and similar events [20]. Safe consumption spaces, also known as supervised injection facilities or overdose prevention sites, consist of medically supervised places where people can use drugs, receive free harm reduction supplies, wrap-around services, and referrals to drug treatment, are innovative interventions that are beginning to be implemented in the USA and have proven to reduce fatal and non-fatal overdoses in Canada and elsewhere [21, 22]. While the current study focused on PWID, both of these interventions could be useful to a broader range of people who use drugs given that fentanyl and its analogues have been detected in heroin that is snorted, in crack cocaine and in counterfeit opioid pills [4, 23, 24].

The high uptake of naloxone trainings among Baltimore SSP clients is largely attributable to the rapid scale up of overdose response trainings offered by the Baltimore City Health Department, which at the time this article was written had trained more than 23,000 people on naloxone use, distributed 18,000 kits, and through these efforts, seen more than 800 overdose reversals. Naloxone programs unfortunately miss those who use drugs alone, which may be sizable (28%) among Baltimore PWID [14]. This signals the urgent need for interventions including safe consumption spaces to enable for rapid on-site responses to overdoses with naloxone to avoid fatalities [21]. The criminalization of drug and paraphernalia possession also poses a substantial barrier to calling EMS in an overdose situation. These data and previous work [13, 25] show that police encounters and fear of arrest are common among SSP clients and may lead to preventable deaths.

Our finding that PWID who used buprenorphine/Suboxone not prescribed to them by a provider had higher odds of non-fatal overdose may be attributable to a number of reasons. One hypothesis is that PWID are using street-acquired buprenorphine that is contaminated with fentanyl; this will require further examination in future studies. Statistics on the origins (diverted versus illegally manufactured) and formulation (e.g., Subutex or Suboxone) of misused buprenorphine in Baltimore are unavailable [26]. Another hypothesis is that this subgroup of PWID may represent a high-risk population of polysubstance users who may increase their dose of heroin to compensate for the opioid-blocking effects of the naloxone-containing formulation (Suboxone) to respond quickly to opioid withdrawal; our findings warrant further investigation. As recommended by the Centers for Disease Control and Prevention, tackling the prescribing practices of providers and reducing levels of diverted prescription medications is a key priority [27]. These data may also highlight a demand for access to effective treatment. Effective linkage of these buprenorphine-misusing PWID to high quality drug treatment could be beneficial. Nationally, finding innovative methods to reduce the gap between the estimated number of people who want treatment and are able to access drug treatment will be critical in stemming the opioid epidemic [28, 29]. This will include supporting a range of treatment options including rehabilitation and detoxification services, medication-assisted treatment, and mental health services as well as point-of-entry harm reduction programs such as syringe exchange programs and supervised safer injection facilities [21, 27].

Finally, we observed that non-fatal overdose was higher among homeless PWID compared to PWID with stable housing, speaking to the importance of attending to the structural vulnerability of this population and their direct impacts on overdose [12, 30]. We found that homelessness was also strongly associated with public injecting (e.g., injecting on the street or in a public bathroom), an association that we have examined further [31]. It is known that homelessness is associated with a range of negative health outcomes among PWID including injection risk behaviors [12, 32]. Further investigation is required to elucidate the complex relationships between homelessness, opioid use, and overdose.

Our study is subject to limitations. Our findings are generalizable to overdose survivors, and thus, our non-fatal overdose prevalence estimates may represent an underestimate of true non-fatal overdose prevalence. Non-fatal overdoses described in this study are not necessarily all attributable to opioids since opioids were not specified in capturing the outcome; however, local surveillance demonstrates that opioids are the dominant cause of drug-related overdose mortality in Baltimore. Many people who test fentanyl-positive may not be aware of the presence of fentanyl contamination in their drugs [5]; the relatively high prevalence of PWID who did not know whether they thought their drugs contained fentanyl was expected and highlights the risks associated with drug use in the context of widespread fentanyl-contamination. This uncertainty highlights the need for community-based fentanyl testing to be made available to PWID.

Our sample consisted primarily of heroin and speedball injectors and may not be generalizable to other subpopulations of PWID. The cross-sectional design of this study and different time periods of available variables do not allow assessment of temporality. Survey data may be subject to recall bias and social desirability. Our study had a moderate sample size, which may have limited statistical power. Given that PWID who are syringe service program clients may have different socio-demographics, health behaviors, and levels of overdose risk compared to non-clients, future studies could examine these associations among non-clients. Finally, patterns and correlates of non-fatal overdose may differ in other urban or rural settings with differing injection drug use epidemiology and differing public health responses in regards to SSP and naloxone training and distribution.

## Conclusions

In conclusion, addressing the opioid overdose epidemic among PWID will require a comprehensive approach that is robust against the rise of synthetic opioids in the illicit drug supply and attends to structural factors often considered beyond the realm of overdose prevention such as homelessness and the types of places where drug use occurs. Innovative strategies that offer a comprehensive approach to meet the needs of drug users are merited. Examples of such strategies include drug checking services, safe drug consumption facilities, the decriminalization of drug and paraphernalia possession, and housing interventions to support existing programs.

## Abbreviations

aOR: Adjusted odds ratio
DEA: Drug Enforcement Administration
PWID: People who inject drugs
SSP: Syringe services program
